# Supplementary material for: Investigating the influence of physiologically relevant hydrostatic pressure on CHO cell batch culture
Source: Sci Rep. 2021 Jan 8;11:162. doi: 10.1038/s41598-020-80576-8 (PMC7794228; doi:10.1038/s41598-020-80576-8)
Supplement: Supplementary file 1 — Supplementary Information 1. [file 41598_2020_80576_MOESM1_ESM.zip › S1/FLEX2/13Jan 30mmHg SS1 is C/Blank.pdf]

===

Acq. Operator : SYSTEM   Seq. Line : 1
Sample Operator : SYSTEM
Acq. Instrument : HPLC-0039   Location : 1
Injection Date : 1/13/2019 5:47:10 PM   Inj : 1
Inj Volume : 100.000 µl
Sequence File : C:\Users\Public\Documents\ChemStation\1\Data\TK_CHO_IgG 2019-01-13 17-45-45 \TK_CHO_IgG.S
Method : C:\Users\Public\Documents\ChemStation\1\Data\TK_CHO_IgG 2019-01-13 17-45-45 \CHO_IgG_LC.M (Sequence Method)
Last changed : 12/4/2018 1:38:41 PM by SYSTEM

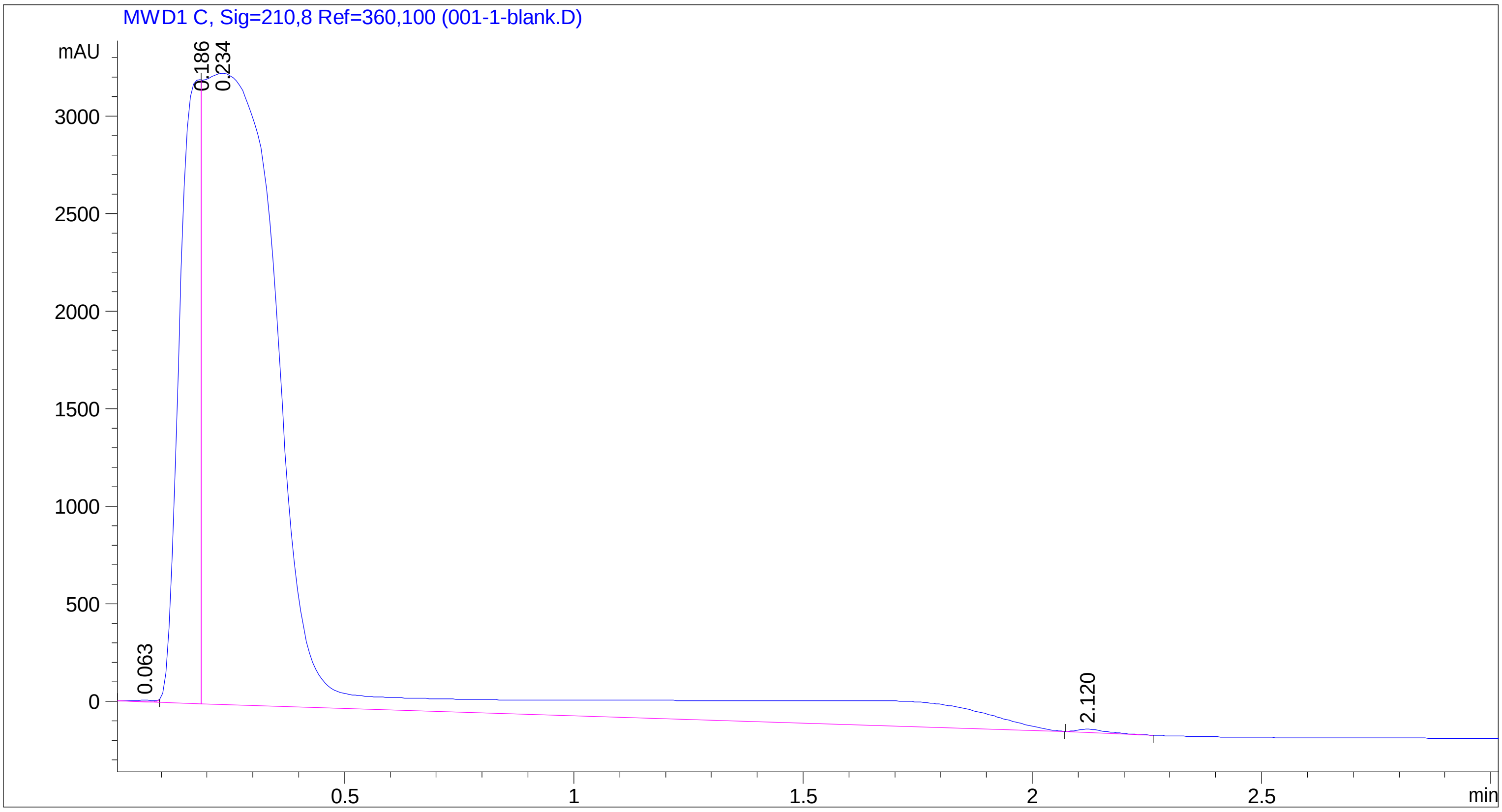

===

Area Percent Report

===

Sorted By : Signal
Multiplier : 1.0000
Dilution : 1.0000
Use Multiplier & Dilution Factor with ISTDs

Signal 1: MWD1 C, Sig=210,8 Ref=360,100

Peak #	RetTime [min]	Type	Width [min]	Area [mAU*s]	Height [mAU]	Area %
1	0.063	BV E	0.0502	24.34789	7.49686	0.0463
2	0.186	VV R	0.0517	1.00306e4	3199.54639	19.0866
3	0.234	VB	0.1990	4.24283e4	3236.15088	80.7343
4	2.120	BBA	0.0647	69.77876	16.07020	0.1328

Totals : 5.25530e4 6459.26432

===

*** End of Report ***
